# Estimating an area-level socioeconomic status index and its association with colonoscopy screening adherence

**DOI:** 10.1371/journal.pone.0179272

**Published:** 2017-06-08

**Authors:** David C. Wheeler, Jenna Czarnota, Resa M. Jones

**Affiliations:** 1 Department of Biostatistics, School of Medicine, Virginia Commonwealth University, Richmond, Virginia, United States of America; 2 Massey Cancer Center, Virginia Commonwealth University, Richmond, Virginia, United States of America; 3 Division of Epidemiology, Department of Family Medicine and Population Health, School of Medicine, Virginia Commonwealth University, Richmond, Virginia, United States of America; University Hospital Llandough, UNITED KINGDOM

## Abstract

Socioeconomic status (SES) is often considered a risk factor for health outcomes. SES is typically measured using individual variables of educational attainment, income, housing, and employment variables or a composite of these variables. Approaches to building the composite variable include using equal weights for each variable or estimating the weights with principal components analysis or factor analysis. However, these methods do not consider the relationship between the outcome and the SES variables when constructing the index. In this project, we used weighted quantile sum (WQS) regression to estimate an area-level SES index and its effect in a model of colonoscopy screening adherence in the Minnesota–Wisconsin Metropolitan Statistical Area. We considered several specifications of the SES index including using different spatial scales (e.g., census block group-level, tract-level) for the SES variables. We found a significant positive association (odds ratio = 1.17, 95% CI: 1.15–1.19) between the SES index and colonoscopy adherence in the best fitting model. The model with the best goodness-of-fit included a multi-scale SES index with 10 variables at the block group-level and one at the tract-level, with home ownership, race, and income among the most important variables. Contrary to previous index construction, our results were not consistent with an assumption of equal importance of variables in the SES index when explaining colonoscopy screening adherence. Our approach is applicable in any study where an SES index is considered as a variable in a regression model and the weights for the SES variables are not known in advance.

## Introduction

Colorectal cancer (CRC) is the second leading cause of cancer-related death in the United States, only secondary to lung cancer [1]. Regular colorectal cancer screening (CRCS) is recommended for average-risk adults ages 50–75 years as it significantly reduces mortality [2–3]. However, CRCS is underutilized with about 35% of age-eligible adults non-adherent to national CRCS recommendations (i.e., having a stool test within a year, sigmoidoscopy within 5 years, and colonoscopy within 10 years) [4].

CRCS adherence is influenced not only by individual-level factors (e.g. age, gender, education, health insurance coverage, barriers to CRCS, etc.) [5–12], but also area-level influences such as socioeconomic status (SES) [13–22]. To date, CRCS studies have examined single measures or discrete sets of SES indicators with the majority reporting significant associations between lower SES and non-adherence [11, 16–24]. SES indices have also been proposed for inclusion in SES-related research [25–27]. There are pros and cons to using single SES variables or composite measures of SES. Using single SES variables can help us understand how a certain aspect of SES is associated with health [28], offer a simple approximation to a deprivation environment, or test a specific hypothesis [29]; however, including multiple correlated SES variables in a traditional regression model may lead to collinearity effects such as regression parameters that are counterintuitive in sign and have inflated variances. In addition, single SES variables cannot fully reflect the whole concept of area SES [30]. Using SES composite measures such as a deprivation index can overcome the aforementioned problems and reduce the dimensionality of the problem.

An SES index or deprivation index is generally constructed using a combination of educational attainment, income, housing, and employment variables and is represented as a composite variable or index with weights for each component [25, 31]. The Townsend and Carstairs deprivation indices used in the United Kingdom also include a variable for percent of no car ownership [25]. Typical approaches to constructing an SES index include creating a sum of z-scores of selected variables [25–27, 32–35], using principal components analysis (PCA) [36], or using factor analysis [26–27, 33]. PCA estimates the weights for each variable in a weighted linear sum of variables to make each component and factor analysis estimates the factor loading that expresses the relationship of each variable to the underlying factor. The variables used to create the SES index using the z-score sum approach may be pre-specified [34–35] or selected using a factor analysis [26–27, 33].

There are drawbacks to these common approaches. For example, combining variable z-scores into a sum places equal weight on each variable, which makes a strong assumption about the relative importance of the variables and may not be appropriate. In addition, using an index with the variable set defined in one geographic area (e.g., a city in the United Kingdom) may not adequately represent SES for a completely different geographic area (e.g., a rural area in the United States). Further, PCA and factor analysis do not consider the relationship between the health outcome and the SES variables when constructing the index. The principal components are constructed based solely on the correlation or covariation pattern among the predictor variables without regard to the outcome variable. Therefore, PCA is not able to perform model selection when building the index. In PCA, the variables with the most variation across the analysis units will receive the most weight [36]. The principal components do not identify a set of SES variables that is associated with a selected health effect as the loadings are the same regardless of the health effect.

Another issue when estimating the association between area SES and CRCS adherence is the selection of spatial scale. It is common in the literature to assume a particular spatial scale is relevant for assessing area-level SES. Many published studies measure area-level SES using variables defined at relatively large geographic scales such as US county [11, 14–19, 21, 23–24, 37], which may obscure effects of area-level SES due to heterogeneous SES within the large geographic areas. Few CRCS-related studies have used smaller geographic scales such as US census tract, block group (a subset of census tract), or ZIP Code [20, 38]. Studies of area-level SES and other health outcomes have calculated SES indices and compared effect estimates at both the census tract- and block group-levels [25, 27, 32], but these studies have not considered spatial scale as a model selection step when assessing the relationship between SES and health outcomes. However, this is an important decision because the estimate of an association can vary depending on the spatial scale used to measure the exposure variable of interest [25, 32]. In addition, recent work demonstrates that different area-level variables are selected into regression models at different spatial scales when spatial scale is considered as a model selection decision [39]. Currently, there is a lack of knowledge about which geographic scale (i.e., census tract vs. block group) is more appropriate for measuring area-level SES as it relates to CRCS adherence.

The objectives of this study were to: (1) estimate an area-level SES index empirically while estimating its association with colonoscopy screening adherence using a novel approach, and (2) determine the spatial scale (i.e., census block group, census tract, or a combination of block group and tract variables–a multiscale SES index) for SES variables that were best for estimating the SES index in a large study of CRCS adherence in Minnesota and Wisconsin.

## Materials and methods

### Setting

As part of the Colorectal Cancer Screening With Improved Shared Decision Making Project (CRCS-WISDM), individual-level data were obtained from the electronic health record (EHR) of Allina Health facilities located in Minnesota counties of the Minneapolis–St. Paul–Bloomington, MN-WI Metropolitan Statistical Area. Allina Health has one of the most comprehensive EHR systems in the nation and had >7 million clinic visits in 2015 [40]. The Virginia Commonwealth University Institutional Review Board approved this study.

### Eligibility

To be eligible for inclusion, patients were: ages 50–75 years (the recommended age for CRCS for average-risk adults) and had at least one primary care-related visit within the past two years at one of 78 Allina Health facilities for each cross-sectional data query (December 2010 –August 2014). Unique patient identification numbers were used to create a retrospective cohort where the most recent data were used for patients who appeared in more than one cross-sectional sample. Overall, 205,755 unique patients were included in the analytic sample.

### Measures and coding

Study measures included colonoscopy, patient-level SES-related variables, area-level SES-related variables as well as Allina Health patients’ addresses. Individual-level variables included gender, age, race/ethnicity, and tobacco use.

#### Colonoscopy adherence

The primary, dichotomous outcome was adherence to national colonoscopy screening recommendations [5], which is the most commonly used CRCS modality [4]. Using EHR data, patients were considered adherent if there was EHR-validated evidence of a colonoscopy in the last 10 years (i.e., September 7, 2004 –August 30, 2014) given the data of the original cross-sectional sampling. The following CPT and ICD 9 codes were used to obtain data on colonoscopy: 1) CPT codes 44388–44394, 44397, 45355, 45378, 45387, 45391, 45392, G0105, G0121; 2) ICD 9 codes 45.22, 45.23, 45.25, 45.42, 45.43.

#### Geocoding

**Fig 1:**
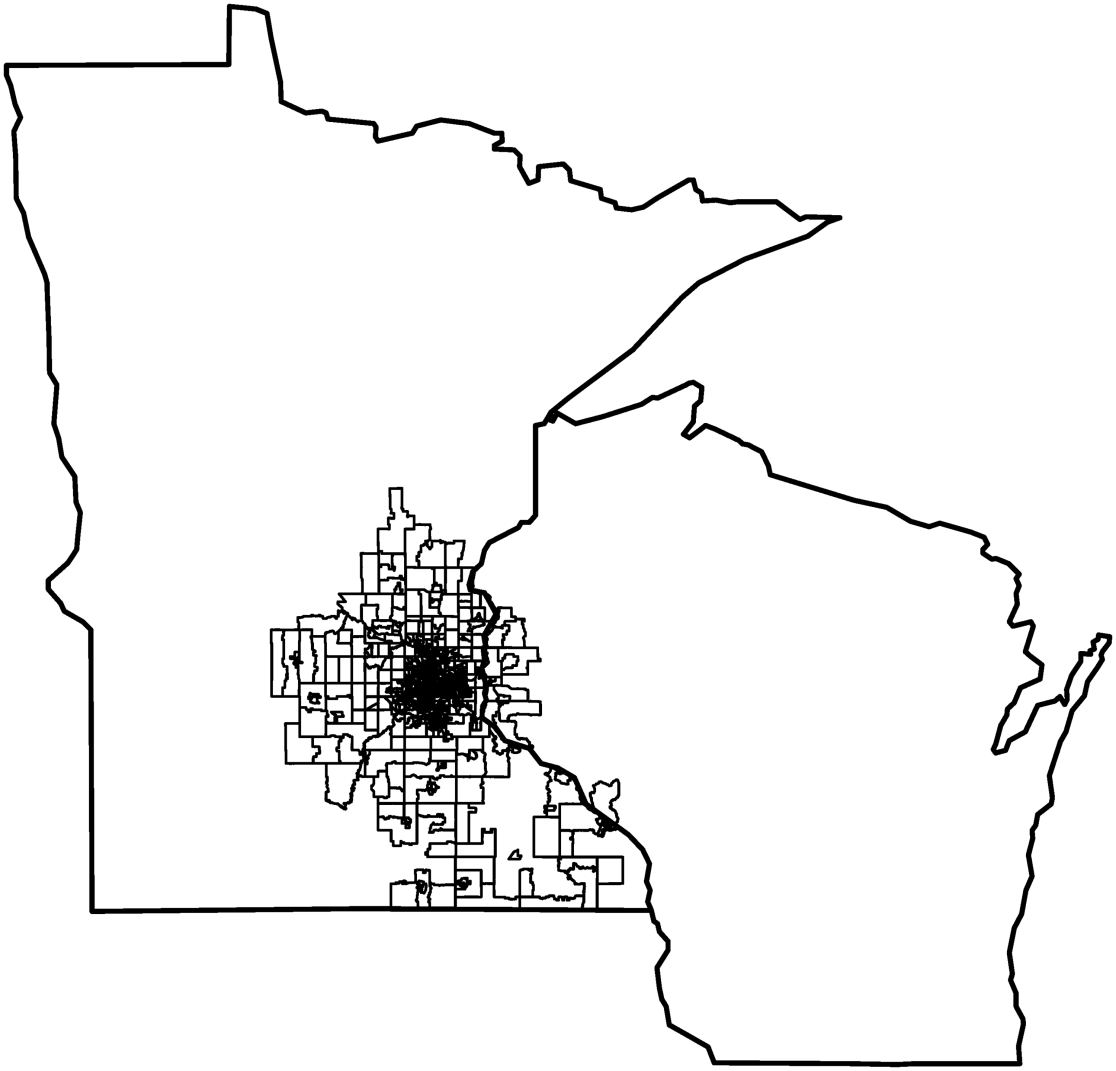
Study area with census tracts that contain study patient residential locations in Minnesota and Wisconsin.

Patients’ most recent residential addresses were geocoded using ESRI Business Analyst Desktop [41] to create an analysis data set with spatial coordinates located in the states of Minnesota and Wisconsin. Geocoding resulted in 93% of addresses matching at the point or street level and the remaining 7% matching at the ZIP Code level. The census tracts in Minnesota and Wisconsin that contain the residential locations of patients included in this study are shown in Fig 1. The spatial coordinates were used to spatially join patients to census block groups and tracts and assign area-level SES variables to patients. Of the 205,755 patients in the analytic sample, 2,049 (1%) were seen in Minnesota Allina facilities but live in Wisconsin.

#### Area-level SES variables

We obtained five-year (2009–2013) estimates of area-level socioeconomic variables from the American Community Survey (ACS) at the census tract- and block group-level for all Minnesota and Wisconsin counties of the Minneapolis–St. Paul–Bloomington, MN-WI Metropolitan Statistical Area. The ACS is administered annually to a nationally representative sample of about three million American households [42]. Selected participants complete an online or paper-based questionnaire for their household and non-responders receive a phone call or personal visit from ACS staff to ensure complete data collection. The questionnaire collects social and economic information such as age, gender, race, income, education, housing, employment and occupations [42].

Due to confidentiality concerns, some ACS variables are not available at the smaller block group scale but are available at the larger census tract scale. We assigned select ACS SES variables to patients using spatial overlay. In addition, we assigned select variables from the 2012 Updated Demographics data from ESRI [43] to patients when geocoding. ESRI produces annual updates to the demographic data based on the 2010 Census and a variety of additional data sources and forecasting methods [43]. The demographic variables available when geocoding were more limited than when using the full ACS downloaded data. The spatial scale of the demographic data variables assigned to patients during geocoding was the block group for point and street matches (93%) and was the tract for ZIP Code matches (7%). More details on the variables used in the analysis are provided below.

### Statistical analysis

To estimate different SES indices and the associated SES effects for colonoscopy adherence, we used weighted quantile sum (WQS) regression. The WQS method [44] is a regression model designed for variable selection in environmental exposure analysis that includes a weighted index of correlated components scored as quantiles. The components in the index are assumed to be reasonable to combine into an index (i.e., all are SES variables) and are constrained to have associations in the same direction with the outcome or have no association. The weights are constrained to sum to 1 and be between 0 and 1, thereby reducing dimensionality and addressing collinearity issues that typically arise with correlated components. The index weights can be empirically determined through the use of bootstrap sampling [44–45] using *B* number of bootstrap samples of size *n* from a training dataset, where *n* is the sample size in the training set. The unknown weights **w** are estimated to maximized the likelihood for *b* = 1 to *B* bootstrap samples for the following model

$${g(\mu )=\beta_{0}+\beta_{1}\left(\sum_{i=1}^{c}w_{i}q_{i}\right)+z^{\prime }\phi |}_{b}$$ (1)

subject to the constraints ${\sum_{i=1}^{c}w_{i}|}_{b}=1$ and 0 ≤ *w*_*i*_ ≤ 1 for *i* = 1 to *c*. In the above equation, *w*_*i*_ represents the weight for the *i*^th^ component *q*_*i*_ and the term $\sum_{i=1}^{c}w_{i}q_{i}$ represents a weighted index for the set of *c* components of interest. The term **z** denotes a vector of covariates determined prior to estimation of the weights, *ϕ* are the coefficients for the covariates in **z**, and *g*(.) is any monotonic and differentiable link function that relates the mean, *μ*, to the predictor variables in the right hand side of the equation. For a binary outcome variable, such as colonoscopy screening adherence, a logit link is assumed for *g*.

The final component weights ${\bar{w}}_{i}$ are calculated as a weighted average of the bootstrap estimates based on the relative strength of the test statistic for *β*_1_ (the parameter estimate for the weighted index) from each bootstrap sample. Using a validation data set, the weighted quantile score is then specificed as $\text{WQS}=\sum_{i=1}^{c}{\bar{w}}_{i}q_{i},$ and the significance of the WQS index is determined using the validation data set and the model

$$g\left(\mu \right)\;=\beta_{0}+\beta_{1}\text{ WQS }+z\prime \phi ,$$ (2)

where exp(*β*_1_) is the odds ratio (OR) associated with a unit increase in the weighted sum of component quartiles. We applied a 50% random split of the analysis data into a training set and validation set to first estimate the SES index weights and then estimate the association of the index with screening adherence.

An advantage of WQS regression compared with other methods for estimating an SES index is that it can estimate the effect of an SES index and the weights for each variable in the SES index while limiting effects of collinearity that arise in traditional regression models. The estimated component weights may be interpreted as measures of relative variable importance in the index. In contrast to PCA or factor analysis, the weights are estimated while considering the association with the outcome variable. In addition, covariates for adjustment are also included in the model that estimates the SES index weights. In other words, all the information contained in the data is used to determine the variables in the SES index that are important. Estimating the SES index weights also avoids the assumption that all variables are equally important in the SES index, which is the assumption of popular indices that sum the z-scores of variables to construct the SES index, such as the Townsend and Carstairs deprivation indices.

We used WQS regression to estimate several SES indices. We estimated an index using the 2012 ESRI demographic data and multiple indices using the 2009–2013 ACS data. We selected a set of SES variables in advance that were available at the block group or census tract scale that we thought could be associated with CRCS adherence. The seven SES variables in the 2012 index included measures of income, poverty, housing tenure, household size, and age dependency, which is defined as the ratio of the young population (<15 years) and the older population (≥65 years) to the working age population (15–64 years). The variables used in the indices are listed in Table 1. We estimated different 2009–2013 SES indices using variables measured at only the block group, only the census tract, and a combination of variables measured at the block group and census tract. The first block group index (BG1) included 10 measures of income, poverty, education, housing tenure, housing rent, and race. The second block group index (BG2) added to BG1 a Gini index of income equality for 11 total variables. The Gini index is 0 with perfect equality and 1 with perfect inequality, and we subtracted this from 1 to match the hypothesized direction (positive) of the variables in the SES index. The first census tract index (CT1) included 15 measures of income, poverty, education, migration, housing tenure, housing rent, health insurance, race, and ethnicity. The second census tract index (CT2) included the variables from BG1 but measured at the census tract instead of at the block group.

**Table 1:** Variables used in different area-level SES indices using census block group and tract ESRI 2012 demographic and ACS 2009–2013 data.

Variable	2012 Index	2009–2013 Block Group Index 1	2009–2013 Block Group Index 2	2009–2013 Tract Index 1	2009–2013 Tract Index 2
Median household income	x	x	x	x	x
Per capita income	x	x	x	x	x
Percent of households not on public assistance		x	x	x	x
Percent of families with children under 18 not in poverty		x	x		x
Percent of population ≥25 years with a bachelor's degree		x	x	x	x
Percent owner occupied housing	x	x	x	x	x
Percent not vacant housing units	x	x	x	x	x
Median gross rent		x	x	x	x
Percent of households with mortgages		x	x	x	x
Percent of population that is white		x	x	x	x
Gini index of income equality			x	x	
Percent of population not in poverty				x	
Percent population ≥1 years in same house as 1 year ago				x	
Percent population 50–74 years in same house as 1 year ago				x	
Percent of people with health insurance				x	
Percent not Hispanic or Latino population				x	
Percent of households not in poverty	x				
Age dependency index	x				
Average household size	x				

There were specific motivations for the set of proposed SES indices. The motivation for the 2012 index was to determine if an easily constructed SES index would provide a relatively good model fit. The 2009–2013 ACS indices were more time intensive to construct due to the required downloading and processing of the ACS data. The motivation for block group index 2 (BG2) was to determine if adding an important variable available at the census tract only improved the model fit of the block group index 1 (BG1). The motivation for tract index 1 (CT1) was to determine if using a larger set of SES variables available at the census tract level would lead to a better fit compared with the block group indices. The motivation for tract index 2 (CT2) was to compare with BG1 to determine which spatial scale resulted in a better model fit. We compared the fit of the different SES index models using the Akaike Information Criterion (AIC). In each of the WQS models, we adjusted for the following individual-level variables when estimating the SES index and effect: age, gender, race, ethnicity, and tobacco use. We fitted the WQS models using the R computing environment with *B* = 100 bootstrap samples. An R package called wqs [46] is available for fitting WQS models.

As a comparison to the approach of using equal weights for each variable in the SES index, we found the best of the fitted WQS models described above according to the AIC and then fixed the weights in the index to be all equal when fitting the model in (Eq 2) above. The AIC for the best WQS model with estimated weights should be significantly lower than the AIC for the WQS model specified with equal weights if our approach is superior to assuming equal importance for all variables in the SES index. In another comparison, we constructed the best WQS model index to match a conventional SES index based on z-scores by replacing the quantiles for the SES variables with z-scores in Eqs (1) and (2). We then compared a model with an index of z-scores and estimated weights to a model with an index of z-scores and fixed and equal weights. Again, if our approach is superior then the model with estimated index weights will have a significantly lower AIC.

## Results

Overall, 52.6% of the sample had a colonoscopy in the last 10 years. According to the AIC, all of the 2009–2013 ACS models fit substantially better than the smaller 2012 model (Table 2). Adding the Gini index of income equality to the block index BG1 meaningfully improved the model fit. Overall, the block group index models fit better than the tract index models. Specifically, the block group models fit substantially better than census tract model CT1 even though the census tract model used more variables in the SES index. The tract model CT2 limited to the variables used in the block group index in model BG1 had worse fit than the model BG1, indicating that using the block group spatial scale led to better fit. The model with the block group SES variables and the census tract Gini index of income equality (BG2) had the best overall fit, demonstrating that a multi-scale SES index can improve on the fit of either a block group or tract only SES index.

**Table 2:** AIC values in the validation dataset for WQS models with different definitions of the SES index.

Model	AIC
2012 Index	137,822
2009–2013 Block Group Index 1	137,772
2009–2013 Block Group Index 2	137,748
2009–2013 Tract Index 1	137,808
2009–2013 Tract Index 2	137,800
2009–2013 Block Group Index 2 with equal weights	137,778
2009–2013 Block Group Index 2 with z-scores	137,754
2009–2013 Block Group Index 2 with z-scores and equal weights	137,780

When the index weights for the best fitting model were fixed to be equal (*w* = 0.091), the AIC increased to 137,778. This is meaningfully higher than the value of 137,748 for the model with weights estimated to be unequal, showing that allowing the variable importance to vary results in better model goodness-of-fit. In fact, using equal variable importance in this model led to a worse goodness-of-fit then the BG1 model, which omitted an important variable (the Gini index). The BG1 model that was clearly inferior to BG2 when the weights were estimated was superior to the best specified BG2 model when it had fixed and equal weights. When using z-scores in place of quantiles for SES variables, the AIC for the model with estimated weights was 137,754 and the AIC for the model with equal weights was 137,780, again demonstrating that estimating the weights led to a better fitting model.

For the best fitting model (BG2), the most heavily weighted variables in the SES index were percent white population, percent owner occupied housing, per capita income, and the Gini index of income equality (Table 3). These variables were also relatively highly weighted in other SES indices. Percent white was the most heavily weighted variable in the BG1, BG2, CT1, and CT2 models. Percent owner occupied housing was the most heavily weighted variable in the model with the 2012 ACS data and the second most heavily weighted variable in the BG1 and CT2 models. Percent white population and the Gini index of income equality were the two highest weighted variables in the index in the CT1 model. Per capita income was the next most highly weighted variable in this model index. The variable percent of population ≥25 years with a bachelor's degree received effectively no weight across the models.

**Table 3:** SES index weights estimated in the training set for variables using census block group and tract ESRI 2012 demographic data and American Community Survey 2009–2013 data.

Variable	2012 Index	2009–2013 Block Group Index 1	2009–2013 Block Group Index 2	2009–2013 Tract Index 1	2009–2013 Tract Index 2
Median household income	0.28	0.13	0.06	0.05	0.15
Per capita income	0.02	0.07	0.14	0.12	0.06
Percent of households not on public assistance		0.02	0.02	0.00	0.01
Percent of families with children under 18 not in poverty		0.02	0.01		0.03
Percent of population ≥25 years with a bachelor's degree		0.00	0.00	0.00	0.00
Percent owner occupied housing	0.29	0.29	0.21	0.04	0.20
Percent not vacant housing units	0.08	0.00	0.00	0.01	0.02
Median gross rent		0.10	0.08	0.02	0.04
Percent of households with mortgages		0.03	0.02	0.00	0.02
Percent of population that is white		0.33	0.27	0.34	0.47
Gini index of income equality			0.17	0.20	
Percent of population not in poverty				0.00	
Percent population ≥1 years in same house as 1 year ago				0.02	
Percent population 50–74 years in same house as 1 year ago				0.01	
Percent of people with health insurance				0.08	
Percent not Hispanic or Latino population				0.08	
Percent of households not in poverty	0.10				
Age dependency index	0.11				
Average household size	0.11				

The SES index for all the models was statistically significantly positively associated with colonoscopy adherence, with odds ratios ranging from 1.10 to 1.17 and p-values < 0.01 (Table 4). The best fitting model had the highest odds ratio (1.17, 95% CI: 1.15–1.19). The covariate reference values were age 70–74 years, female, white, non-Hispanic, and never used tobacco. Screening adherence was significantly positively associated with ages 55–69 years, white race, non-Hispanic ethnicity, and former tobacco use. Current tobacco use and age 50–54 years were significantly inversely related to screening adherence in all models.

**Table 4:** Odds ratios estimated in the validation set for variables in models with different SES indices based on ESRI 2012 demographic data and American Community Survey 2009–2013 data.

Variable	2012 Index	2009–2013 Block Group Index 1	2009–2013 Block Group Index 2	2009–2013 Tract Index 1	2009–2013 Tract Index 2
Intercept	-	-	-	-	-
Age 50–54	0.649*	0.650*	0.649*	0.652*	0.651*
Age 55–59	1.342*	1.345*	1.344*	1.348*	1.347*
Age 60–64	1.228*	1.228*	1.228*	1.231*	1.230*
Age 65–69	1.375*	1.375*	1.375*	1.377*	1.376*
Male	0.980	0.979	0.979	0.982^	0.980
Race Null	0.689*	0.700*	0.700*	0.699*	0.700*
Race Black	0.728*	0.754*	0.756*	0.747*	0.746*
Race Asian	0.613*	0.633*	0.632*	0.636*	0.637*
Race American Indian	0.837	0.853	0.854	0.844	0.843
Race Native Hawaiian	0.565*	0.576*	0.577*	0.579*	0.577*
Ethnicity Null	0.725*	0.731*	0.729*	0.731*	0.732*
Ethnicity Hispanic	0.629*	0.641*	0.641*	0.638*	0.638*
Tobacco Use Null	0.057*	0.057*	0.057*	0.056*	0.056*
Tobacco Use Quit	1.035^	1.034^	1.032^	1.029^	1.031^
Tobacco Use Passive	0.872	0.875	0.873	0.869	0.869
Tobacco Use Yes	0.565*	0.565*	0.564*	0.558*	0.560*
SES Index	1.106*	1.154*	1.172*	1.149*	1.117*

*p-value < 0.01

^p-value < 0.05

**Fig 2:**
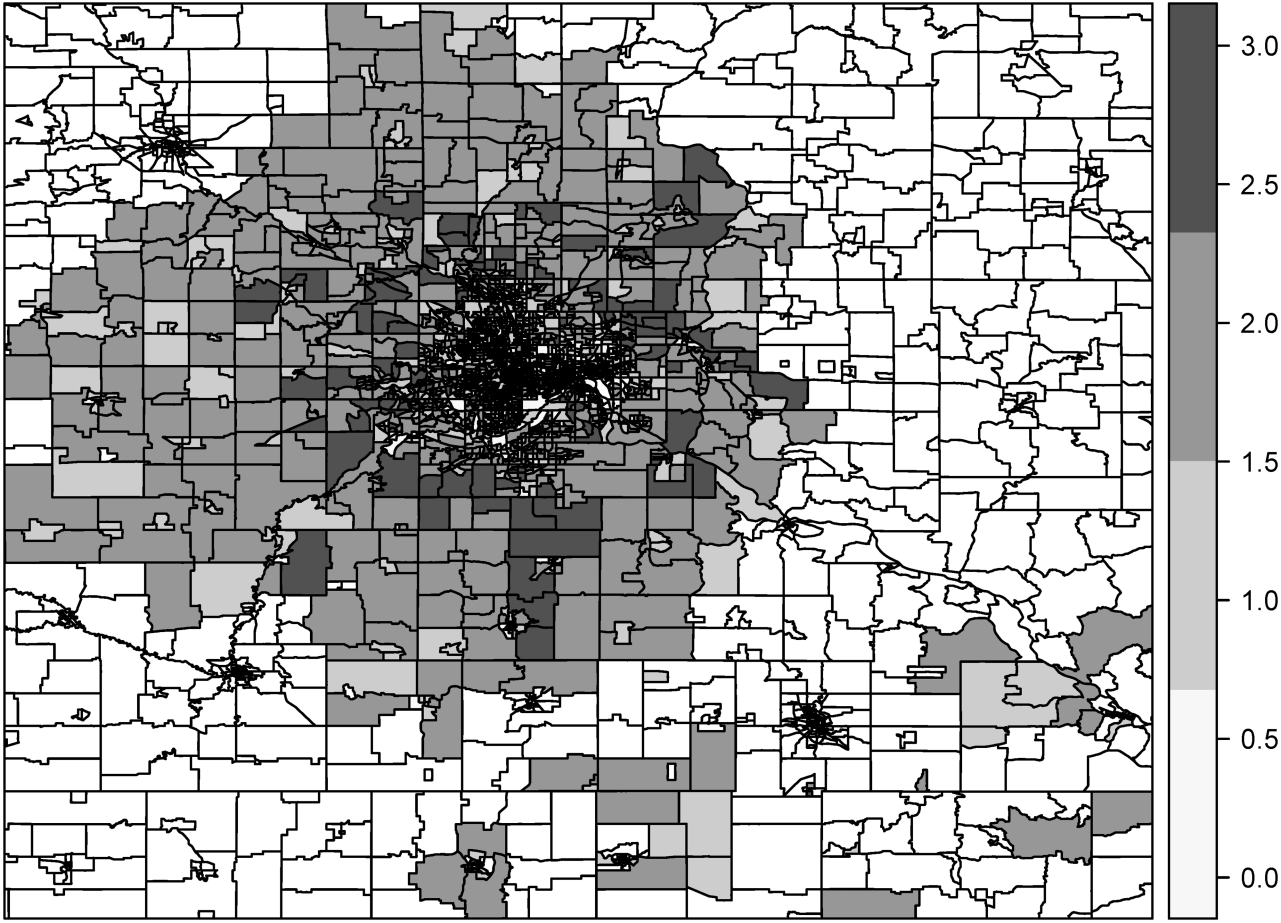
SES index for block groups estimated with block group model 1 (BG1) in the training set.

The estimated SES index for census tracts from model CT2 (not shown) and for block groups from model BG1 (Fig 2) show spatial variation in area-level SES with higher values on the periphery of Minneapolis-St. Paul. The block group index has more spatial heterogeneity than the tract index given the smaller spatial unit.

## Discussion

In this paper, we estimated an area-level SES index and its association with colonoscopy using WQS regression in a large study of patients in Minnesota and Wisconsin. Our results demonstrate that it is feasible to simultaneously estimate an area SES index and its association with an outcome using WQS regression. We found a statistically significant relationship between area-level SES and colonoscopy adherence. Certain variables in the index, such as home ownership and race, were estimated to be more important than others. Some variables, like education, were estimated to have effectively no weight in the index. These results show that for this study assuming equal weights for the variables in the SES index, which is done in the typical approach when summing z-scores to create an index [25–27, 32–35], lead to a worse fitting model. Instead of assuming a strict definition of the SES index, we learned from the data how to represent the SES index.

There are several advantages of using WQS regression to estimate an area-level SES index. The WQS regression method was designed to estimate the effect of a combination of many variables, identify the individual variables most strongly associated with a health outcome, and adjust for risk factors. The method is designed to accommodate highly correlated data that create collinearity issues with traditional regression methods. It identifies the important variables in the index through the estimated index weights, in contrast to z-score sums that treat the variables as equal in importance. Simulation studies show that WQS regression has high specificity and adequate sensitivity in identifying important variables in regression models [44, 47]. In simulation studies based on pairwise correlations of 11 chemicals in NHANES (2005–2008), WQS regression had greater accuracy in identifying the 7 of 11 truly important chemicals (i.e., chemicals set to be related to the outcome) correctly as the correlation of the exposures and the outcome increased from that observed (range of 0.03 to 0.08) to three times that observed [44]. It also showed an improvement in specificity over traditional regression and popular shrinkage methods (e.g., lasso, adaptive lasso and elastic net). In another simulation study, WQS regression had good sensitivity and specificity in all exposure scenarios for both continuous and binary outcome variables [47] and had higher specificity than penalized regression models. The studies show that WQS regression tends to place negligible weight on components with no correlation with the outcome. In addition, WQS regression can be used for a large number of SES variables, as the method has been successfully used to model disease risk related to a mixture of 27 chemicals [45, 47].

Placing the SES index construction into a modeling framework allows one to explore different model specifications and select the model with the best goodness-of-fit. We capitalized on this modeling approach to investigate different spatial scales for the SES index and found that a spatial scale of block group provided a better fit to the data than a spatial scale of census tract. Using a modeling-based approach to select the spatial scale is superior to assuming a particular spatial scale for the analysis and leads to better model fit [39]. In our case, we found that a multi-scale SES index provided the best fit by combining an important variable available only at the census tract with a set of variables available at the block group. This consideration of spatial scale is similar in spirit to the recent development of algorithms to select the spatial scale for each area-level covariate in linear regression models [39]. Similar to avoiding the assumption of equal variable importance in the index, by treating spatial scale of SES variables as a model selection problem we avoid making the assumption that one spatial scale is correct for all SES indices.

Our study has several limitations. First, misclassification of colonoscopy adherence may exist. For example, data for colonoscopy tests done at facilities outside the Allina Health system may not be captured in the Allina Health EHR. Also, for those in the first three of six cross-sectional samples, data on colonoscopy were only available in the EHR for 7 to 9 years. This misclassification would result in people being considered non-adherent while they are actually adherent to CRCS guidelines. Second, we used patients’ most recent address to geocode. Thus, the addresses that are geocoded may not reflect the addresses where patients lived when they obtained CRCS. However, migration within MN is quite low; for example, only 3% of 65–74 year olds move to another address in the state [48]. Third, this study used the census tract and block group as a proxy of neighborhood, which may not realistically reflect meaningful neighborhoods or communities in all cases. Fourth, not all patient-level variables and area-level indices that have been cited as relevant to the association between SES and health outcomes were available for our analysis [25–27, 32]. Therefore, it is possible that the observed effects of area-level SES and individual colonoscopy behaviors could be affected by unmeasured confounders. Fifth, we were not able to differentiate between people at high-risk for CRC and those at average-risk. Thus, both screening and diagnostic colonoscopy were included in the main outcome. However, adherence was defined in this study as having a colonoscopy in the last 10 years, which aligns with national recommendations for average-risk adults [3]. Lastly, the study sample only included Allina Health patients who had a primary care visit in the Twin Cities metropolitan area in MN in the past two years of each cross-sectional sampling date. Thus, it may not completely represent the general population or those who do not access care.

Our approach of using WQS regression is applicable in any study where an SES index is considered as a variable in a regression model and the weights for the SES variables are not known in advance. While we used this approach for estimating area-level SES indices, it could also be used to estimate an individual-level SES index if the required variables were available. The feasibility of applying this method in other studies is aided by the presence of an R package [46].
